# Chemical chaperones ameliorate neurodegenerative disorders in Derlin-1-deficient mice via improvement of cholesterol biosynthesis

**DOI:** 10.1038/s41598-022-26370-0

**Published:** 2022-12-17

**Authors:** Takashi Sugiyama, Naoya Murao, Hisae Kadowaki, Hideki Nishitoh

**Affiliations:** 1 Laboratory of Biochemistry and Molecular Biology, Faculty of Medicine, University of Miyazaki, 5200 Kihara, Kiyotake, Miyazaki, 889-1692 Japan; 2 Department of Neurology, Faculty of Medicine, University of Miyazaki Hospital, 5200 Kihara, Kiyotake, Miyazaki, 889-1692 Japan; 3 Division of Respirology, Rheumatology, Infectious Diseases, and Neurology, Department of Internal Medicine, Faculty of Medicine, University of Miyazaki, 5200 Kihara, Kiyotake, Miyazaki, 889-1692 Japan; 4 Frontier Science Research Center, University of Miyazaki, 5200 Kihara Kiyotake, Miyazaki, 889-1692 Japan

**Keywords:** Biochemistry, Neuroscience, Neurology

## Abstract

There are no available therapies targeting the underlying molecular mechanisms of neurodegenerative diseases. Although chaperone therapies that alleviate endoplasmic reticulum (ER) stress recently showed promise in the treatment of neurodegenerative diseases, the detailed mechanisms remain unclear. We previously reported that mice with central nervous system-specific deletion of *Derlin-1*, which encodes an essential component for ER quality control, are useful as models of neurodegenerative diseases such as spinocerebellar degeneration. Cholesterol biosynthesis is essential for brain development, and its disruption inhibits neurite outgrowth, causing brain atrophy. In this study, we report a novel mechanism by which chemical chaperones ameliorate brain atrophy and motor dysfunction. ER stress was induced in the cerebella of Derlin-1 deficiency mice, whereas the administration of a chemical chaperone did not alleviate ER stress. However, chemical chaperone treatment ameliorated cholesterol biosynthesis impairment through SREBP-2 activation and simultaneously relieved brain atrophy and motor dysfunction. Altogether, these findings demonstrate that ER stress may not be the target of action of chaperone therapies and that chemical chaperone-mediated improvement of brain cholesterol biosynthesis is a promising novel therapeutic strategy for neurodegenerative diseases.

## Introduction

Neurodegenerative diseases, including Parkinson’s disease, amyotrophic lateral sclerosis (ALS) and Alzheimer’s disease, manifest as progressive impairment of the structure or function of a particular group of neurons^1^. The cause of these diseases has not yet been clarified, although studies have postulated that they results from the accumulation of abnormal proteins and inflammation^1,2^. Recent studies have also reported the contribution of metabolic disorders to neurodegenerative diseases^3^, especially Alzheimer’s disease, which was found to be strongly related to abnormal lipid metabolism^4^. Although the brain represents only 2% of total body weight, it contains approximately 20% of the body’s cholesterol, which accounts for approximately 20–30% of the brain weight^5,6^. Therefore, it is not surprising that abnormal cholesterol biosynthesis has been implicated in the development of neurodegenerative diseases.

Disturbance of endoplasmic reticulum (ER) proteostasis is known to be involved in the development of some neurodegenerative diseases, including spinocerebellar degeneration and ALS^7^. The ER is an organelle responsible for protein folding and lipid synthesis; however, pathological ER stress occurs when unfolded proteins accumulate in the ER. Cells possess mechanisms to reduce ER stress through the unfolded protein response (UPR), such as protein refolding and ER-associated degradation (ERAD)^8,9^, and excessive ER stress causes apoptosis and contributes to the development of neurodegenerative diseases^7,10,11^. However, the UPR exerts diverse effects other than inducing apoptosis. The pathogenesis of neurodegenerative diseases cannot be explained solely in terms of neuronal cell death due to the disruption of ER proteostasis, indicating the need for further analysis of the involvement of mechanisms other than neuronal cell death. We recently discovered a relationship between ER dysfunction and neurological disorders by generating mice with central nervous system (CNS)-specific deletion of *Derlin-1* (*Derl1*) or *Derlin-2* (*Derl2*), which encode vital components of the ERAD and ER stress-induced preemptive quality control (ERpQC) systems^12^, and observed impaired neurite outgrowth due to disrupted cholesterol synthesis in neurons and progressive brain atrophy, primarily in the cerebellum^13^. The most crucial feature of neurodegenerative diseases is brain atrophy, which corresponds to the observed symptoms. Neuronal loss is believed to be important in brain atrophy; however, a decrease in the number of neurons alone does not results in sufficient brain atrophy to cause symptoms. Hence, the phenotypes of CNS-specific Derlin-deficient mice, such as brain atrophy following impaired neurite outgrowth, are consistent with the pathology of neurodegenerative diseases, and these mice can be considered a model of neurodegenerative diseases, especially spinocerebellar degeneration.

Although treatments based on the molecular mechanisms of neurodegenerative diseases have not yet been established, chaperone therapies that alleviate ER stress have shown promise in ameliorating the pathophysiology of neurodegenerative diseases^14^. A representative chemical chaperone, 4-phenylbutyric acid (4-PBA), is known to ameliorate the phenotypes of mouse models of neurodegenerative diseases; however, the detailed mechanisms, including the direct involvement of ER stress, have not been clarified^15,16^. In the present study, we focused on brain atrophy in our mouse model of neurodegenerative diseases to explore the effects of chemical chaperones and the mechanisms related to their ability to alleviate pathology.

## Results

### Chemical chaperones do not alleviate ER stress in the cerebella of CNS-specific Derlin-1-deficient mice

**Figure 1:**
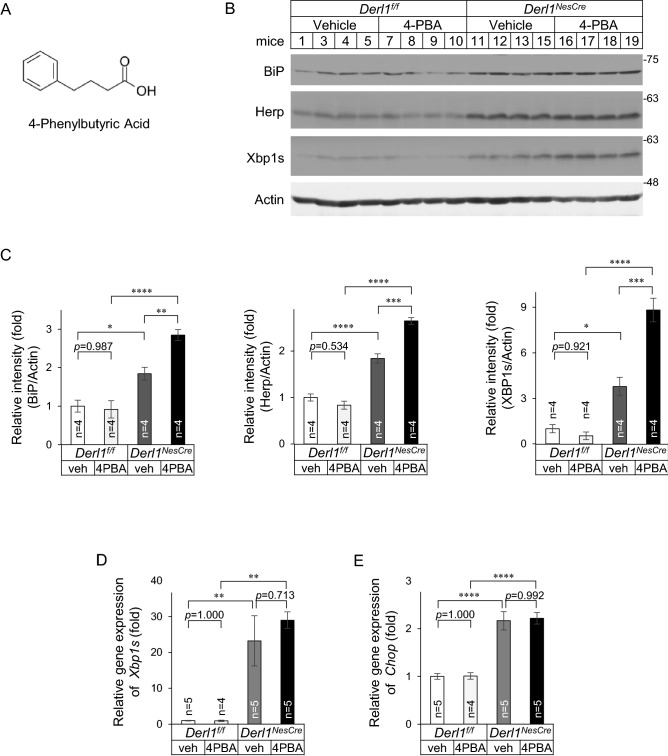
Chemical chaperone does not alleviate ER stress in the cerebellum of CNS-specific Derlin-1-deficient mice. (**A**) The structural formula of 4-PBA. (**B**–**E**) Vehicle or 4-PBA was administered via intraperitoneal injections once a day from P14 to P28 of age. (**B**) Expression of BiP, Herp, and Xbp1s in the cerebellum of *Derl1*^*f/f*^ and *Derl1*^*NesCre*^ mice at P28 after vehicle or 4-PBA treatment. Whole tissue lysates from the cerebellum were analyzed by IB with the indicated antibodies. Mice of 1, 3–5, 7–13, 15, and 19 are female, and those of 16–18 are male. Uncropped original immunoblotting images are included in Fig. S1, with cropped areas highlighted with colored boxes. (**C**) BiP, Herp, Xbp1s, and actin band intensities were measured, and the amount of BiP, Herp, or Xbp1s was normalized to that of actin. Data are shown as the fold change relative to the average band intensity in *Derl1*^*f/f*^ mice treated with vehicle. (**D**, **E**) Expression of *Xbp1s* (**D**) and *Chop* (**E**) genes in the cerebellum of *Derl1*^*f/f*^ and *Derl1*^*NesCre*^ mice at P28 after vehicle or 4-PBA treatment. The gene expression levels were analyzed by qPCR. The mRNA expression of the indicated genes was normalized to that of *S18*. Data are shown as the fold change relative to the value in vehicle-treated *Derl1*^*f/f*^ mice. All mice except two 4-PBA-treated *Derl1*^*NesCre*^ mice were females. Data information: bar graphs are presented as mean ± SEM. *P < 0.05; **P < 0.01; ***P < 0.001; ****P < 0.0001; one-way ANOVA followed by Tukey’s test (**C**–**E**).

CNS-specific Derlin-1-deficient (*Derl1*^*NesCre*^) mice exhibit concurrent impairment of neurite outgrowth and ER stress induction in cerebellar regions where atrophy is observed^13^. ER stress is attenuated by chemical chaperones, among which 4-PBA (Fig. 1A) can easily cross the blood–brain barrier^17^. 4-PBA has been confirmed to exert a therapeutic effect in mouse models of neurodegenerative diseases such as Alzheimer’s disease and Parkinson’s disease^15,16^. Therefore, we administered 4-PBA intraperitoneally to *Derl1*^*NesCre*^ mice and their controls (*Derl1*^*f/f*^ mice) from postnatal day 14 (P14) to P28 and investigated the changes in ER stress in cerebellar tissue in vivo. We observed that the protein expression of BiP, Herp, and Xbp1s, which was analysed by immunoblotting (IB) (Fig. 1B, C), and the mRNA expression of *Xbp1s* and *Chop,* which was analysed by quantitative real-time PCR (qPCR) (Fig. 1D, E), were upregulated in *Derl1*^*NesCre*^ mice, which is consistent with our previous report^13^. However, the upregulation of these molecules was not attenuated by 4-PBA administration. The discrepancy in results between the previous and current experiments may have been due to differences in the concentration of 4-PBA acting on neurons in vitro and in vivo or the presence or absence of supporting cells around neurons. In any case, our findings suggest that Derlin-1 deficiency induced ER stress in mouse brain tissues and that this ER stress was not attenuated by the intraperitoneal administration of the chemical chaperone under our experimental conditions.

### Chemical chaperones restore the activation of sterol regulatory element binding protein 2 in Derlin-1-deficient mice

**Figure 2:**
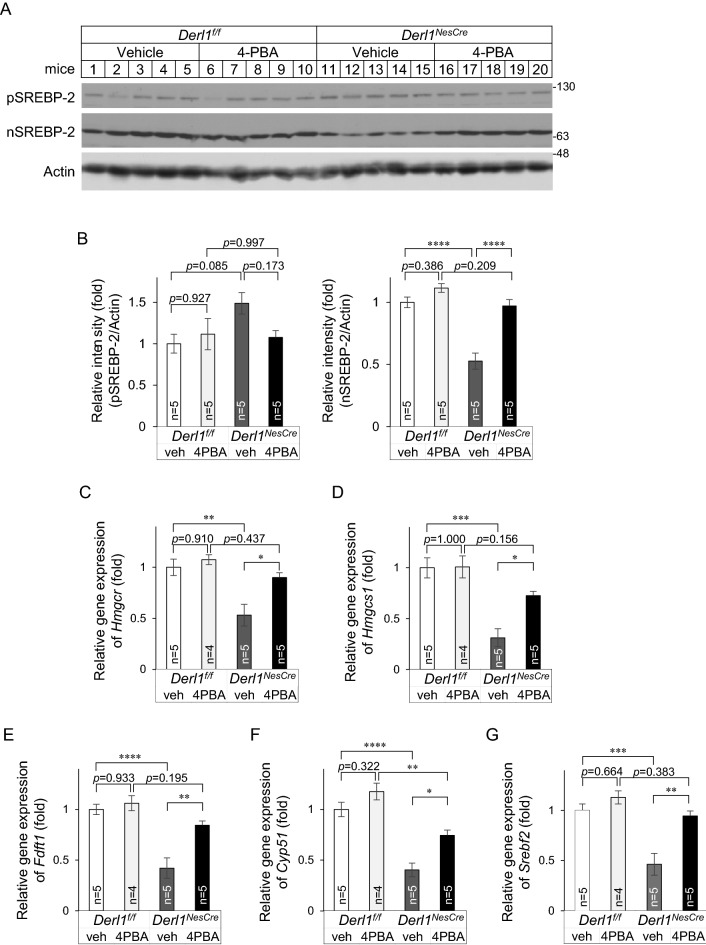
Chemical chaperones restore the activation of SREBP-2 in Derlin-1-deficient mice. (**A**–**G**) Vehicle or 4-PBA was administered via intraperitoneal injections once a day from P14 to P28 of age. (**A**) Expression of precursor (p) SREBP-2 and nuclear (n) SREBP-2 in the cerebellum of *Derl1*^*f/f*^ and *Derl1*^*NesCre*^ mice at P28 after vehicle or 4-PBA treatment. Whole tissue lysates from the cerebellum were analyzed by IB with SREBP-2 and actin antibodies. Mice of 1–15, 19, and 20 are female, and those of 16–18 are male. Uncropped original immunoblotting images are included in Fig. S2, with cropped areas highlighted with colored boxes. (**B**) pSREBP-2, nSREBP-2, and actin band intensities were measured, and the amount of pSREBP-2 or nSREBP-2 was normalized to that of actin. Data are shown as the fold change relative to the average band intensity in *Derl1*^*f/f*^ mice treated with vehicle. (**C**–**G**) Expression of cholesterol biosynthesis-related genes (C, *Hmgcr*; D, *Hmgcs1*; E, *Fdft1*; F, *Cyp51*; G, *Srebf2*) in the cerebellum of *Derl1*^*f/f*^ and *Derl1*^*NesCre*^ mice at P28 after vehicle or 4-PBA treatment. The gene expression levels were analyzed by qPCR. The mRNA expression of the indicated genes was normalized to that of *S18*. Data are shown as the fold change relative to the value in vehicle-treated *Derl1*^*f/f*^ mice. All mice except two 4-PBA-treated *Derl1*^*NesCre*^ mice were females. Data information: bar graphs are presented as mean ± SEM. *P < 0.05; **P < 0.01; ***P < 0.001; ****P < 0.0001; one-way ANOVA followed by Tukey’s test (**B**–**G**).

Previously, we reported that Derlin-1 deficiency induces not only ER stress but also the inhibition of cholesterol synthesis in the brain^13^. Cholesterol is essential for brain development and function^18^. The synthesis of cholesterol is tightly regulated by the activation of the ER membrane-anchored transcription factor sterol regulatory element binding protein 2 (SREBP-2)^19^. In the brains of *Derl1*^*NesCre*^ mice, the cholesterol synthesis pathway is impaired, and exogenous expression of an active form of SREBP-2 alleviates the decrease in the length of neurites of primary cultured neurons^13^. To examine whether treatment with a chemical chaperone affects cholesterol biosynthesis, we analysed the expression of SREBP-2 in the cerebella of 4-PBA-treated mice by IB. Our results showed that 4-PBA treatment increase the level of the active form of SREBP-2 [nuclear (n)SREBP-2] in the cerebella of *Derl1*^*NesCre*^ mice (Fig. 2A, B). However, the level of precursor (p)SREBP-2 in the cerebella of *Derl1*^*NesCre*^ mice tended to decrease upon 4-PBA administration (Fig. 2A, B). Consistent with the results of IB, we established that the impaired nuclear translocation of SREBP-2 in Purkinje cells in the cerebella of *Derl1*^*NesCre*^ mice was ameliorated by 4-PBA administration (Fig. S3). These data suggest that 4-PBA administration partially, but not completely, reverses the reduction in SREBP-2 activation. Furthermore, along with SREBP-2 activation, 4-PBA treatment increased the expression of key cholesterol biosynthesis genes regulated by SREBP-2, specifically, *Hmgcr*, *Hmgcs1*, *Fdft1*, *Cyp51*, and *Srebf2*, which encodes SREBP-2^20^ (Fig. 2C–G). Total cerebellar cholesterol levels were reduced in *Derl1*^*NesCre*^ mice but tended to be increased by 4-PBA treatment (Fig. S4). Overall, 4-PBA treatment improved the impairment of cholesterol biosynthesis without ameliorating ER stress in *Derl1*^*NesCre*^ mice.

### Chemical chaperones ameliorate changes in brain size and motor impairment

**Figure 3:**
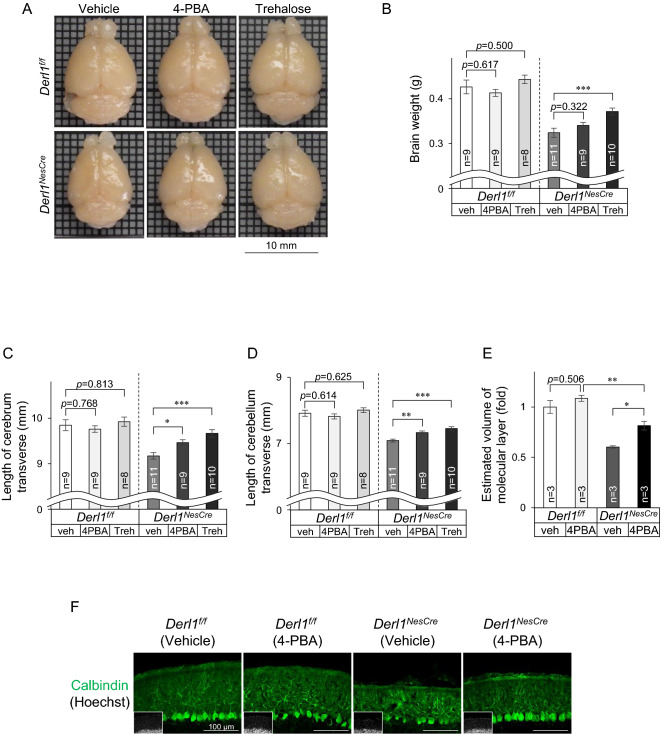
Chemical chaperone ameliorates brain atrophy. (**A**–**F**) Vehicle or chemical chaperone was administered via intraperitoneal injections once a day from P14 to 8 weeks of age. All mice were males (**A**–**F**). (**A**) Representative gross brain images between vehicle or chemical chaperone (4-PBA or trehalose)-treated *Derl1*^*NesCre*^ mice at 8 weeks of age. Scale bar, 10 mm. (**B**) Comparison of brain weight between vehicle or chemical chaperone (4-PBA or trehalose) treated *Derl1*^*NesCre*^ mice and their respective control mice. (**C**, **D**) Comparison of brain length between vehicle- or chemical chaperone (4-PBA or trehalose) treated *Derl1*^*NesCr*e^ mice and their respective control mice. Transverse diameters on photographs of the cerebrum (**C**) and cerebellum (**D**) were measured, respectively. (**E**) Volumetric analysis of the molecular layer of the cerebellum in *Derl1*^*NesCre*^ mice and their respective control mice at 8 weeks of age after vehicle or 4-PBA treatment. Estimated volumes of the cerebellum were calculated according to Cavalieri’s principle using manually measured areas. Data are shown as the fold change relative to the value of *Derl1*^*f/f*^ mice. (**F**) Immunohistochemical analysis of cerebellar molecular layer volume using anti-Calbindin-stained sections from *Derl1*^*NesCre*^ and their respective control mice at 8 weeks of age after vehicle or 4-PBA treatment. Data information: bar graphs are presented as mean ± SEM. *P < 0.05; **P < 0.01; ***P < 0.001; one-way ANOVA followed by Dunnett’s test (**B**–**D**); one-way ANOVA followed by Tukey’s test (**E**).

We next investigated the impact of 4-PBA treatment on brain atrophy in *Derl1*^*NesCre*^ mice (Fig. 3A). The brain weight of *Derl1*^*NesCre*^ mice tended to be increased by 4-PBA treatment, although not significantly (Fig. 3B). The reduction in the size of the cerebrum and cerebellum in *Derl1*^*NesCre*^ mice was ameliorated by 4-PBA treatment (Fig. 3C, D). Moreover, the increase in the volume of the cerebellum tended to be similar to the increase in the transverse diameter of the cerebellum (Fig. 3D, S5, S6). In the cerebellum, the improvement was particularly obvious in the molecular layer, which contains Purkinje cells (Fig. 3E, F, S7). 4-PBA functions not only as a chemical chaperone but also as a histone deacetylase (HDAC) inhibitor^21^. To explore whether the observed phenotypic improvement was due to the function of 4-PBA as an HDAC inhibitor, we used trehalose, another chemical chaperone that has no HDAC inhibitory function. We detected further drastic improvements in brain weight and cerebrum and cerebellum sizes after trehalose treatment (Fig. 3A–D). These findings suggest that chemical chaperone activity is important in ameliorating brain atrophy caused by impairment of ER function in the brain.

**Figure 4:**
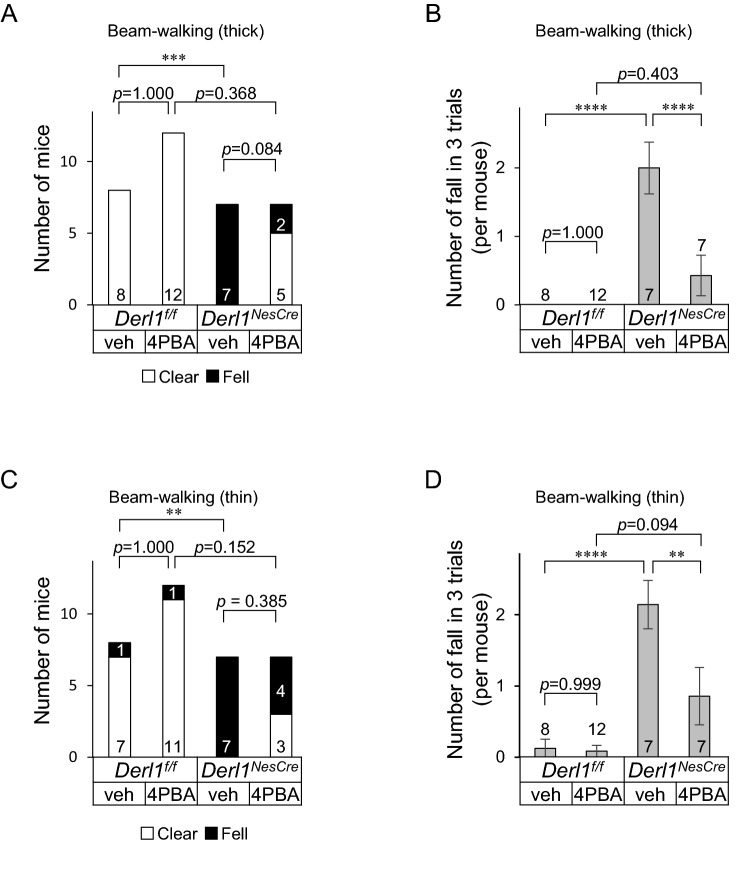
Chemical chaperone improves motor dysfunction. (**A**–**D**) Beam-walking test was performed after vehicle or 4-PBA was administered by ad libitum access to each solution from P14 to approximately 13 weeks of age. Mice walked three trials per day on a thick beam on the first day (**A**, **B**) and a thin beam on the second day (**C**, **D**). (**A**, **C**) Comparison of the number of mice that fell off one or more times in three trials (Fell) and the number that safely reached the platform in three of three trials (Clear). (**B**, **D**) Comparison of the number of falls in three trials per mouse. Data information: bar graphs are presented as mean ± SEM. *P < 0.05; **P < 0.01; ***P < 0.001; ****P < 0.0001; Fisher’s exact test followed by Holm’s test (**A**, **C**); one-way ANOVA followed by Tukey’s test (**B**, **D**).

Finally, we investigated whether 4-PBA treatment alleviates motor dysfunction in *Derl1*^*NesCre*^ mice. Similar numbers of male and female mice that had been treated with 4-PBA until approximately 13 weeks of age were subjected to the beam-walking test (Fig. S8). *Derl1*^*NesCre*^ mice exhibited impaired motor coordination in the beam-walking test, which is consistent with our previous report (Fig. 4A–D)^13^. Mice treated with 4-PBA showed a significant decrease in the number of falls in three trials on both thick and thin beams (Fig. 4B, D). In general, motor function inversely correlates with body weight in mice, with lighter mice having better motor function^22^. *Derl1*^*NesCre*^ mice were slightly lighter than control mice, and 4-PBA treatment did not have any effect of their body weight (Fig. S9). Altogether, these data suggest that 4-PBA treatment alleviates motor dysfunction in *Derl1*^*NesCre*^ mice.

In conclusion, chemical chaperone treatment does not alleviate the impairment of ER quality, at least not completely, in *Derl1*^*NesCre*^ mice but ameliorates brain atrophy and motor coordination impairment through the activation of cholesterol biosynthesis.

## Discussion

ER stress is currently believed to play a role in the development of neurodegenerative diseases, and chemical chaperone therapy has been developed to reduce ER stress. However, in the present study, administration of a chemical chaperone in mice lacking Derlin-1, an ERAD and ERpQC component, did not reduce ER stress, and enhancement of the cholesterol metabolic pathway through SREBP-2 activation was essential for phenotypic improvement. CNS-specific Derlin-2-deficient (*Derl2*^*NesCre*^) mice also exhibit impaired postnatal brain development and motor control deficits due to impaired cholesterol synthesis in neurons^13^. Administration of 4-PBA ameliorated brain atrophy in to *Derl2*^*NesCre*^ mice, although not as much as in *Derl1*^*NesCre*^ mice (Fig.S10). These observations indicate that chemical chaperone therapy, while useful, does not have a simple target of action.

Two issues were not addressed in this study. First, 4-PBA reduced ER stress in primary cultured Derlin-1-deficient neurons but did not improve neurite outgrowth^13^; however, intraperitoneal administration of 4-PBA to *Derl1*^*NesCre*^ mice did not ameliorate ER stress but increased brain size. Although 4-PBA is a small-molecule compound that efficiently crosses the blood–brain barrier^17^, the dose administered to the mice in this study may not have allowed the amount of the drug needed to reduce ER stress to reach the brain. Alternatively, the discrepancy in results between the previous in vitro study and current in vivo experiment may have been due to differences in the presence or absence of supporting cells around neurons. Nevertheless, an interesting observation was that cholesterol biosynthesis was improved and that some phenotypes were alleviated. Astrocytes are also known to produce cholesterol in the brain, and adult neurons depend on astrocytes for cholesterol supply^18^. Therefore, it is possible that the phenotypic improvement observed in our experiments was due to improved cholesterol biosynthesis in not only neurons but also astrocytes. Nonetheless, our results emphasize that the cholesterol biosynthesis pathway, not the simple UPR, may be a therapeutic target for ER stress-related neurodegenerative diseases. The second issue is that the mechanism by which 4-PBA promotes SREBP-2 activation is unclear. SREBP-2 activation is tightly controlled in a cholesterol concentration-dependent manner in the ER^19,23^. When cellular cholesterol is depleted, the ER transmembrane protein SREBP cleavage-activating protein (Scap) escorts SREBP-2 from the ER to the Golgi apparatus, where it is sequentially cleaved by Golgi-resident site-1 and site-2 proteases. Following cleavage, the amino-terminal form of SREBP-2 translocates to the nucleus, where it induces the transcription of cholesterol biosynthesis-related genes. However, when excess cholesterol accumulates in the ER membrane, Scap interacts with insulin-induced gene (Insig)-1 and Insig-2, inhibiting Scap/SREBP-2 transport to the Golgi apparatus. Insig-1 is ubiquitinated by the E3 ubiquitin ligase gp78 and degraded by the ERAD pathway during cholesterol depletion^24,25^. Because Derlin-1 interacts and coordinates with gp78 in the ERAD complex^26,27^, 4-PBA treatment may induce the degradation of Insigs stabilized in Derlin-1-deficient cells and enhance the translocation of Scap/SREBP-2 to the Golgi apparatus. The Scap/SREBP-2 complex is degraded by the proteasome in cells treated with 25-hydroxyvitamin D^28^. It is possible that 4-PBA treatment may stabilize Scap and SREBP-2, which are degraded by the Derlin-1-independent degradation pathway. Although further studies are needed to elucidate the precise molecular mechanisms by which chemical chaperones ameliorate impaired cholesterol biosynthesis in the brains of Derlin-1-deficient mice, there are several drugs that may exploit these mechanisms. The mechanism of chaperone therapy in the treatment of the metabolic disease Fabry disease, a genetic disorder in which the enzymatic activity of α-galactosidase A is reduced, involves the binding of migalastat to unstable α-galactosidase A and migalastat-mediated promotion of α-galactosidase A transport to lysosomes, promoting enzyme activity^29^. Sodium phenylbutyrate, a salt of 4-PBA, can also inhibit the elevation of blood ammonia levels in urea cycle disorders resulting from the excretion of glutamine from the body by binding to it^30^. Regarding neurodegenerative diseases, an expectorant ambroxol that increases β-glucocerebrosidase activity and decreases α-synuclein accumulation is attempted as a chaperone therapy for Parkinson's disease^31^. Similar to 4-PBA, these pharmaceutical agents may promote the activation of SREBP-2 and cholesterol synthesis in the atrophied brain. However, the detailed molecular mechanism remains unknown and needs to be further investigated.

It is expected that chaperone therapies could be studied in several clinical treatments in the future, and the traditional role of chaperones, i.e., to help transport molecules, is also important in neurodegenerative diseases. Because cholesterol does not cross the blood–brain barrier, compounds and strategies that promote cholesterol synthesis in the brain might emerge as novel therapeutic approaches for neurodegenerative diseases.

## Methods

### Animals

All mice used in this experiment were raised under specific pathogen-free conditions and housed under a 12 h/12-h light/dark cycle with free access to food and water or 4-PBA solution. Details regarding *Derl1*^*f/f*^ mice, *Derl2*^*f/f*^ mice, and mice expressing Cre recombinase driven by the nestin promoter have been described in previous reports^13,32^. These mice were intercrossed to generate *Derl1*^*NesCre*^ mice and *Derl2*^*NesCre*^ mice. Both male and female mice were used. All mice experiments were approved by the Animal Research Committee of the University of Miyazaki in accordance with institutional guidelines. The experiments were conducted according to institutional guidelines. All efforts were made to minimize animal suffering and reduce the number of animals used. ARRIVE guidelines were followed in all animal experiments.

### Tissue preparation for biochemical analysis

Mice were sacrificed by cervical dislocation, and brains were rapidly dissected for IB and real-time qPCR. The cerebellum was frozen immediately on dry ice and stored at − 80 °C.

### Tissue preparation for immunofluorescence

Mice were deeply anesthetized by intraperitoneal injection of a 4 mg/kg midazolam/0.3 mg/kg medetomidine/5 mg/kg butorphanol mixture, and transcardially perfused with phosphate buffered saline (PBS) followed by 4% paraformaldehyde (PFA) in PBS. Brains were dissected and post-fixed overnight in the same fixative at 4 °C. Fixed brains were incubated in 15% sucrose solution at 4 °C overnight followed by incubation in 30% sucrose solution at 4 °C overnight. Brains were then cut into two pieces along the midline, and each half was embedded in optimal cutting temperature compound (Tissue Tek; Sakura Finetek; 4583) and stored at − 80 °C. Embedded frozen brains were serially sectioned in the coronal plane at 40-mm thickness using a freezing microtome (Leica Microsystems; CM3050S), and every sixth section was sequentially transferred to 6-well plates in PBS for subsequent immunohistochemical staining.

### Immunohistochemistry

The brain sections were washed with PBS and incubated in blocking solution (PBS containing 3% FBS and 0.1% Triton X-100) for 1 h at room temperature (RT) followed by overnight incubation at 4 °C with the indicated primary antibody diluted in blocking solution. Sections were washed thrice with PBS and incubated for 2 h at RT with secondary antibody diluted in blocking solution. After a final wash with PBS, the sections were mounted on glass slides with Immu-Mount (Thermo Scientific; 9990402). Immunofluorescence images were obtained using a confocal laser microscope (Leica Microsystems; TSC-SP8) or fluorescence microscope (KEYENCE; BZ-9000) and processed using Adobe Photoshop Elements (Adobe). Nuclei were counter stained using bisbenzimide H33258 fluorochrome trihydrochloride solution (Hoechst; nacalai tesque, 19173-41). Antibodies are listed in Supplementary Table S1.

### Volumetric analysis and SREBP-2 nuclear translocation analysis

Volumetric analyses were conducted using every sixth 40-μm coronal half brain section stained with NeuN. The areas of each brain region were measured using ImageJ software (National Institutes of Health), and volume (V) was calculated as V = ΣA × i × d according to Cavalieri’s principle, where A is the sum of target areas in each section, i is the interval between the sections, and d is the section thickness. Fold changes between *Derl1*^*NesCre*^ mice and their respective controls were calculated as measures of regional brain atrophy. SREBP-2 nuclear translocation analysis was conducted using coronal half cerebellum sections from *Derl1*^*NesCre*^ and their respective controls treated with vehicle or 4-PBA from P14 to 8 weeks of age were stained using Hoechst (nacalai tesque, 19173-41) and anti-SREBP-2 antibody (St John's Laboratory, STJ115016). All sections were visualized by confocal laser microscopy (Leica Microsystems; TSC-SP8). Ten cerebellar Purkinje cells were analyzed from three unrelated animals per experimental group. The intensity of SREBP-2 staining in nuclear areas (nSREBP-2) and cytoplasmic areas (pSREBP-2) were measured using Image J software (National Institutes of Health). The relative fold intensity of nSREBP-2 staining per pSREBP-2 staining was calculated.

### Immunoblotting

Whole-cell lysates were prepared by homogenizing brain and other tissues for 60 s in lysis buffer (20 mM Tris–HCl pH 7.5, 150 mM NaCl, 5 mM EGTA, and 1% Triton X-100) supplemented with 5 μg/mL leupeptin (nacalai tesque; 43449-62) on ice using a Micro Smash (TOMY; MS-100) (4500 rpm, 4 °C). Whole-cell lysates were resolved by sodium dodecyl sulfate–polyacrylamide gel electrophoresis (SDS-PAGE) and blotted onto polyvinylidene fluoride (PVDF) membranes. After blocking with 5% skim milk in TBS-T (50 mM Tris–HCl pH 8.0, 150 mM NaCl, and 0.05% Tween-20), the membranes were probed with the indicated antibodies, and immunolabeling was detected using an enhanced chemiluminescence (ECL) system. Antibodies are listed in Supplementary Table S1. Band intensity was measured using ImageQuant TL (GE Healthcare).

### qPCR analysis

Total RNA was isolated from the cerebellum at P28 using RNAiso Plus (Takara Bio; 9109) or the RNeasy Plus Mini Kit (QIAGEN; 74104) and reverse-transcribed using RevaTra Ace qPCR RT Master Mix with gDNA Remover (TOYOBO; FSQ-301). qPCR was performed using SYBR Green PCR Master Mix (KAPA BIOSYSTEMS; KK4602) and a StepOnePlus Real-Time PCR System (Applied Biosystems). Expression levels were normalized to the expression of *S18* mRNA. The primer sequences are shown in Supplementary Table S2.

### Cholesterol assay

Total cholesterol content in the cerebellum was measured using a colorimetric assay kit according to the manufacturer’s instructions (Cell Biolabs; STA-384).

### Beam-walking test

The apparatus used for the mice consisted of a 1-m-long steel pipe suspended 30 cm above the floor and connected to a safe platform. In each test, a mouse was placed on the pipe at the starting end, and the number of mice that fell off or safely reached the platform one or more times of three trials was recorded. *Derl1*^*NesCre*^ mice and their respective control mice were subjected to three trials per day on a thick beam on the first day and on a thin beam on the second day.

### Chemical chaperone administration

Intraperitoneal injections of 200 mg/kg of 4-PBA (MERCK; 820986) were performed once a day from P14 to P28 for IB and qPCR. For measuring brain size, 200 mg/kg of 4-PBA and 500 mg/kg of trehalose (nacalai tesque; 34413-92) were administered by intraperitoneal injections once a day from P14 to 8 weeks of age. In the cerebellar cholesterol content measurement and behavioral test, 10 mg/ml of 4-PBA (Sigma-Aldrich; P21005) solution was administered ad libitum from P14 to each experimental timing. Because long-term intraperitoneal injection causes significant stress to mice, we switched to oral administration for the experiments in which we performed behavioural tests and measured cerebellar cholesterol levels, which took more than one and a half months.

### Quantification and statistical analysis

All data are presented as mean ± standard error. One-way ANOVA and Fisher’s exact test followed by post hoc tests were conducted to compare three or more group means. All statistical analyses were performed using the EZR software version 1.30^33^. *P* < 0.05 (two-tailed) was considered to be significant for all tests.

## Supplementary Information

Supplementary Figures.

Supplementary Tables.

## Data Availability

The datasets generated during and analyzed during the current study are available from the corresponding author on reasonable request.
