# Supplementary material for: Chemical chaperones ameliorate neurodegenerative disorders in Derlin-1-deficient mice via improvement of cholesterol biosynthesis
Source: Sci Rep. 2022 Dec 17;12:21840. doi: 10.1038/s41598-022-26370-0 (PMC9759528; doi:10.1038/s41598-022-26370-0)
Supplement: Supplementary file 1 — Supplementary Figures. [file 41598_2022_26370_MOESM1_ESM.pdf]

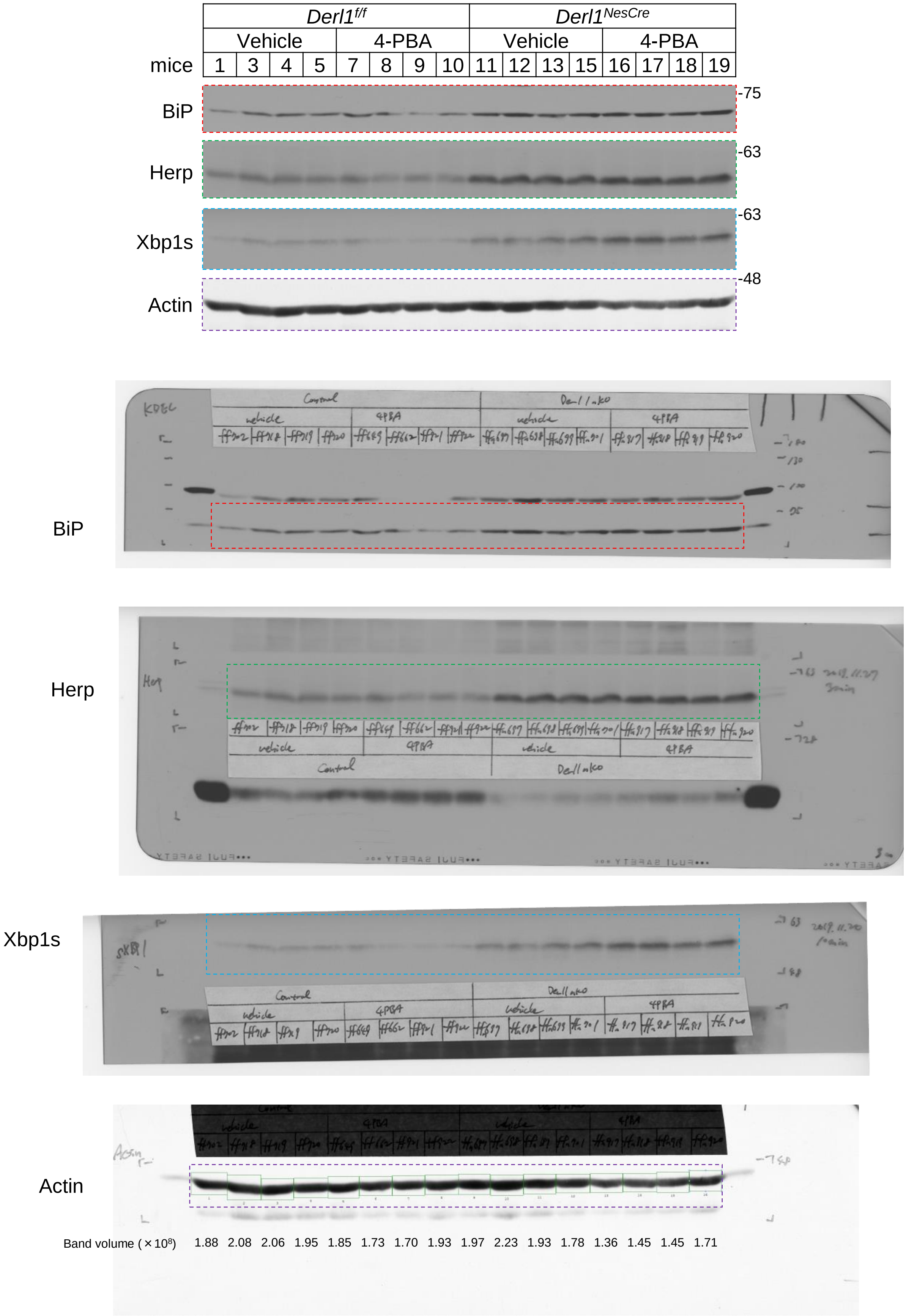

Figure S1. Related to Figure 1. Uncropped gel.
Full length blots of whole tissue lysates from the cerebellum (shown as cropped images in Figure 1B).

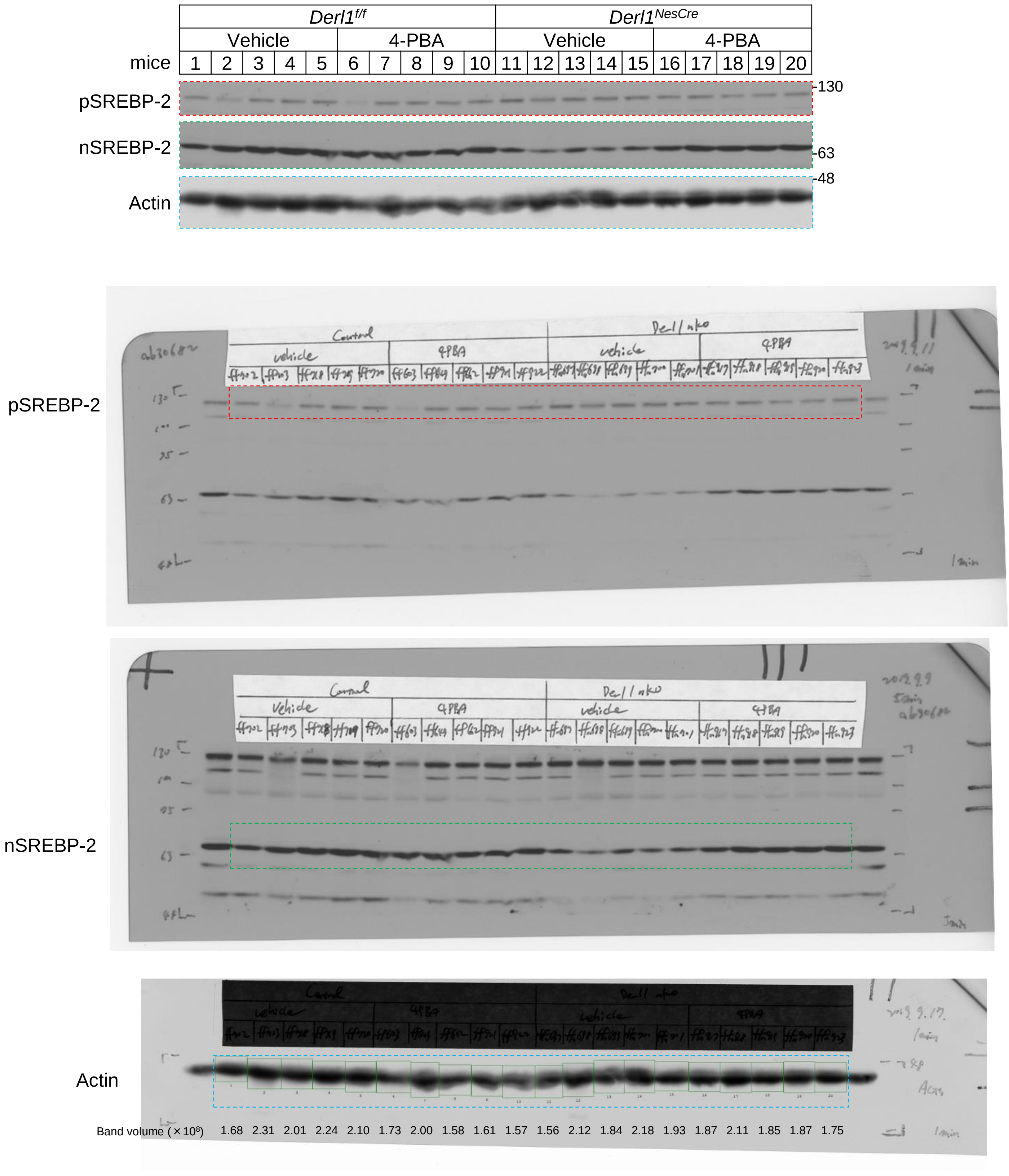

Figure S2. Related to Figure 2. Uncropped gel.
Full length blots of whole tissue lysates from the cerebellum (shown as cropped images in Figure 2A).

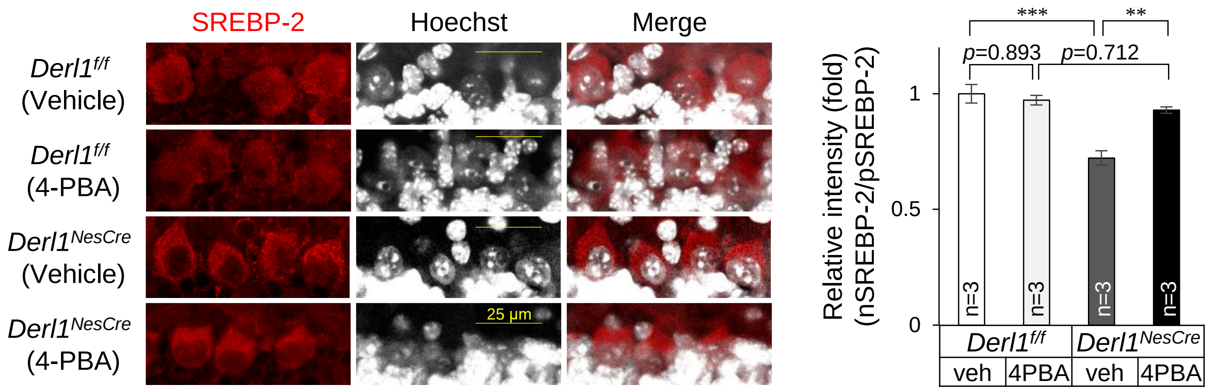

Figure S3. Related to Figure 2. SREBP-2 nuclear translocation analysis of cerebellar Purkinje cells.
Vehicle or 4-PBA was administrated intraperitoneal injections once a day from P14 to 8 weeks of age. *Derl1*^{NesCre} mice and their respective control mice at 8 weeks of age were used for immunohistochemical analysis of cerebellar Purkinje cells in Hoechst and anti-SREBP-2 antibody-stained sections. (left) Representative immunofluorescence images of Purkinje cells. (right) The relative fold intensity of SREBP-2 staining in nuclear area (nSREBP-2) per cytoplasmic area (pSREBP-2). Data information: bar graphs are presented as mean ± SEM. $^{**}P < 0.01$; $^{***}P < 0.001$; one-way ANOVA followed by Tukey's test.

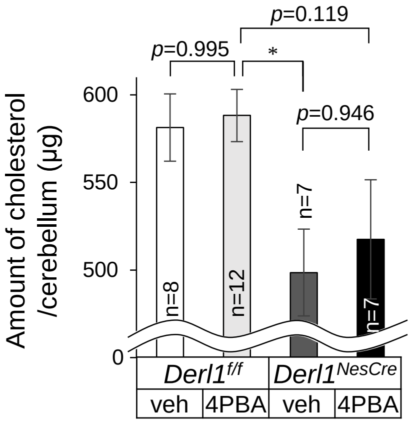

Figure S4. Related to Figure 2. Quantification of total cholesterol in the cerebellum.
Vehicle or 4-PBA was administered by ad libitum access to each solution from P14 to 16 weeks of age. The mice used consisted of approximately equal numbers of males and females (See Table S8). Quantification of the total amount of cholesterol in the cerebellum of *Derl1*^{NesCre} mice and their respective control mice at 16 weeks of age after vehicle or 4-PBA treatment. Data information: bar graphs are presented as mean ± SEM. *P < 0.05; one-way ANOVA followed by Tukey's test.

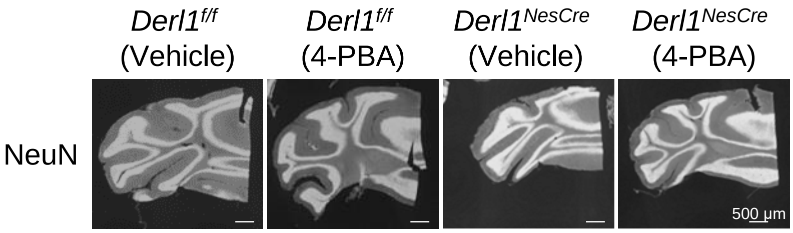

Figure S5. Related to Figure 3, S6 and S7. Representative immunofluorescence images of the entire cerebellum.

Vehicle or 4-PBA was administrated intraperitoneal injections once a day from P14 to 8 weeks of age. Immunohistochemical analysis of whole cerebellum, cerebellar molecular layer and cerebellar granular layer volume using anti-NeuN-stained serial sections from *Derl1*^{NesCre} mice and their respective control mice at 8 weeks of age.

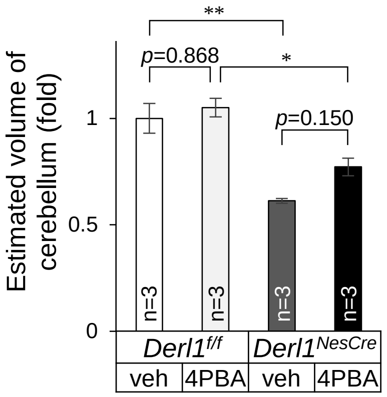

Figure S6. Related to Figure 3. Change of volumes of whole cerebellum.
Vehicle or 4-PBA was administrated intraperitoneal injections once a day from P14 to 8 weeks of age. Volumetric analysis of the whole volume of the cerebellum in *Derl1*^{NesCre} mice and their respective control mice at 8 weeks of age. Estimated volumes of the cerebellum were calculated according to Cavalieri's principle using manually measured areas. Data are shown as the fold change relative to the value of *Derl1*$^{f/f}$ mice. Data information: bar graphs are presented as mean ± SEM. $^*P < 0.05$; $^{**}P < 0.01$; one-way ANOVA followed by Tukey's test.

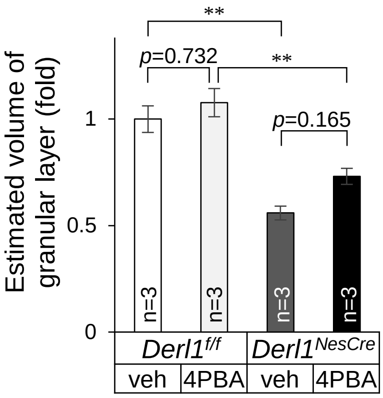

Figure S7. Related to Figure 3. Change of volumes of granular layer.
Vehicle or 4-PBA was administrated intraperitoneal injections once a day from P14 to 8 weeks of age. Volumetric analysis of the granular layer volume of the cerebellum in *Derl1*^{NesCre} mice and their respective control mice at 8 weeks of age. Estimated volumes of the granular layer were calculated according to Cavalieri's principle using manually measured areas. Data are shown as the fold change relative to the value of *Derl1*$^{f/f}$ mice. Data information: bar graphs are presented as mean ± SEM. **P < 0.01; one-way ANOVA followed by Tukey’s test.

	Derl1$^{f/f}$		*Derl1*^{NesCre}		
	Vehicle	4-PBA	Vehicle	4-PBA	p value
Number	8	12	7	7	
Male / Female	5/3	8/4	4/3	5/2	1.000
Age of weeks (SEM)	12.99 (0.33)	13.31 (0.30)	13.14 (0.28)	13.54 (0.46)	0.737

Figure S8. Related to Figure 4 and S4. Background of Beam-walking test and cholesterol assay.
Comparison of sexuality and age between vehicle and 4-PBA treated *Derl1*^{NesCre} mice and their respective control mice. Data information: one-way ANOVA followed by Tukey's test and Fisher's exact test.

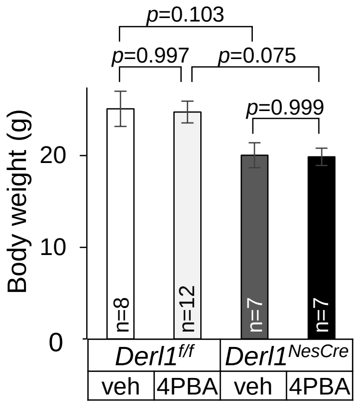

Figure S9. Related to Figure 4. Background of Beam-walking test.
Comparison of body weight between vehicle and 4-PBA treated *Derl1*^{NesCre} mice and their respective control mice. Data information: bar graphs are presented as mean ± SEM. one-way ANOVA followed by Tukey's test.

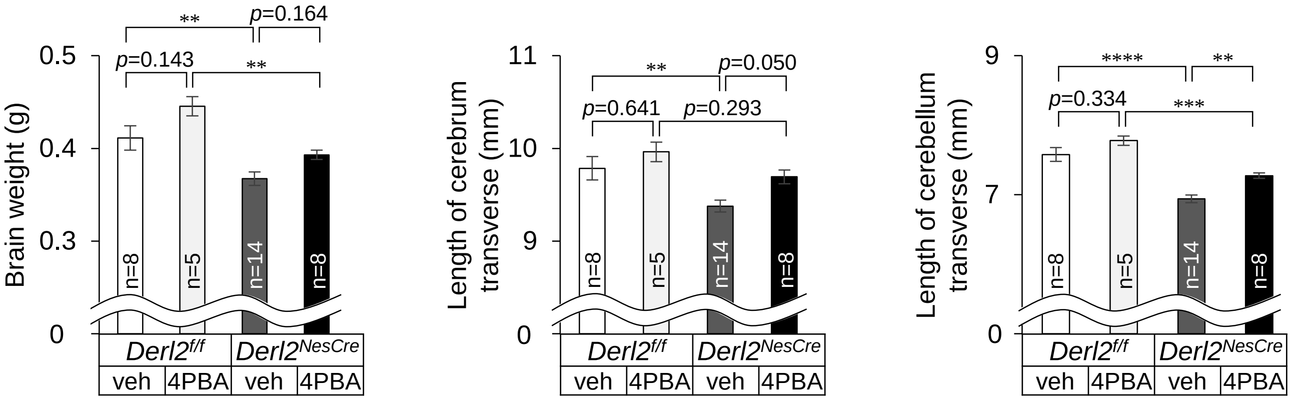

Figure S10. Chemical chaperone ameliorates brain atrophy in *Derl2*^{NesCre} mice.

Vehicle or 4-PBA was administrated intraperitoneal injections once a day from P14 to 8 weeks of age. Volumetric analysis of the whole volume of the cerebellum in *Derl2*^{NesCre} mice and their respective control mice at 8 weeks of age. Comparison of brain weight (left) and brain length (middle and right) between vehicle or 4-PBA treated *Derl2*^{NesCre} mice and their respective control mice. Data information: bar graphs are presented as mean ± SEM. **P < 0.01; ***P < 0.001; ****P < 0.0001; one-way ANOVA followed by Tukey's test.
