# Supplementary material for: Chemical chaperones ameliorate neurodegenerative disorders in Derlin-1-deficient mice via improvement of cholesterol biosynthesis
Source: Sci Rep. 2022 Dec 17;12:21840. doi: 10.1038/s41598-022-26370-0 (PMC9759528; doi:10.1038/s41598-022-26370-0)
Supplement: Supplementary file 2 — Supplementary Tables. [file 41598_2022_26370_MOESM2_ESM.pdf]

Table S1. Antibodies for immunoblotting and immunofluorescence

Antibodies	Source	Identifier
Mouse-anti-Actin	Sigma-Aldrich	A4700; RRID: AB_476730
Rabbit-anti-SREBP2	Abcam	ab30682; RRID: AB_779079
Rabbit-anti-SREBF2	St John's Laboratory	STJ115016; RRID: N/A
Mouse-anti-NeuN	Millipore	MAB377; RRID: AB_2298772
Mouse-anti-Calbindin D-28k	Sigma-Aldrich	c-9848; RRID: AB_476894
Mouse-anti-KDEL	Enzo Life Sciences	ADI-SPA-827, RRID: AB_10618036
Rabbit-anti-Herp	[34]	N/A
Rabbit-anti-XBP-1s	Cell signaling Technology	12782; RRID: AB_2687943
Anti-rabbit IgG, HRP-linked antibody	Cell signaling Technology	7074; RRID: AB_2099233
Anti-Mouse IgG, HRP-linked antibody	GE Healthcare	NA931; RRID: AB_772210
CF®488A, Donkey Anti-Mouse IgG (H+L), Highly Cross-Adsorbed	Biotium	20014; RRID: AB_10561327
CF®555, Donkey Anti-Rabbit IgG (H+L), Highly Cross-Adsorbed	Biotium	20038; RRID: AB_10558011

Table S2. Primers for quantitative real-time PCR

Species	Gene name	Forward	Reverse	Reference
Mouse	*Xbp1s*	GGTCTGCTGAGTCCGCAGCAGG	GGTCTGCTGAGTCCGCAGCAGG	[35]
Mouse	*Chop*	CCACCACACCTGAAAGCAGAA	AGGTGAAAGGCAGGGACTCA	[35]
Mouse	*Srebf2*	TGGACCTCACGGGGGACTC	CTCTCCCACTTGATTGCTGACA	N/A[#)]
Mouse	*Hmgcs1*	AAATGCCAGACCTACAGGTGG	ATGCTGCATGTGTGTCCCA	Harvard Primer Bank ID: 148747293c3
Mouse	*Fdft1*	AGAAGGACCGACAAGTGCTG	CCCAGTCCTGTTTGGAGGTC	[13]
Mouse	*Cyp51*	AACGAAGACCTGAATGCAGAAG	GTGGGCTATGTTAAGGCCACT	Harvard Primer Bank ID: 71061450c3
Mouse	*Hmgcr*	AGAGCGAGTGCATTAGCAAAG	GATTGCCATTCCACGAGCTAT	Harvard Primer Bank ID: 160358777c2
Mouse	*S18*	TCCAGCACATTTTGCGAGTA	CAGTGATGGCGAAGGCTATT	[36]

#) Forward and reverse primers for *Srebf2* were newly designed by the authors with NCBI Primer-BLAST.

<Suplementary References>

34 Fujisawa, T. *et al.* A novel monoclonal antibody reveals a conformational alteration shared by amyotrophic lateral sclerosis-linked SOD1 mutants. *Annals of neurology* **72**, 739-749, doi:10.1002/ana.23668 (2012).

35 Liu, J. *et al.* Inflammation Improves Glucose Homeostasis through IKKβ-XBP1s Interaction. *Cell* **167**, 1052-1066.e1018, doi:10.1016/j.cell.2016.10.015 (2016).

36 Hattori, K. *et al.* ASK1 signalling regulates brown and beige adipocyte function. *Nature communications* **7**, 11158, doi:10.1038/ncomms11158 (2016).
